# Patient and care partner perceptions of the new Multispecialty Interprofessional Team primary care‐based memory clinics across four Canadian provinces

**DOI:** 10.1002/alz70858_100406

**Published:** 2025-12-25

**Authors:** Linda Lee, Loretta M Hillier, Michael Lee, Tejal Patel, Carrie A McAiney

**Affiliations:** ^1^ McMaster University, Kitchener, ON, Canada; ^2^ Schlegel‐UW Research Institute on Aging, Waterloo, ON, Canada; ^3^ MINT Memory Clinic, Kitchener, ON, Canada; ^4^ Geras Centre for Aging Research, Hamilton, ON, Canada; ^5^ University of Waterloo, Waterloo, ON, Canada; ^6^ Schlegel‐UW Research Institute for Aging, Waterloo, ON, Canada

## Abstract

**Background:**

Multispecialty Interprofessional Team (MINT) Memory Clinics manage dementia and other memory disorders at a primary care‐level. Established in Ontario, with over 100 clinics, this care model has recently expanded to four other Canadian provinces. This study describes patient and care partner experience with this new care model.

**Methods:**

Satisfaction surveys were distributed in 14 newly established clinics, across four Canadian provinces, to patients and care partners following their clinic assessment. Survey completion was anonymous. Five‐point rating scales (“strongly disagree” to “strongly agree”) were used to rate appointment timeliness, quality of explanations, satisfaction with time spent, understanding of symptoms/ condition, willingness to recommend the clinic to others, value of the clinic in addition to regular primary care; overall satisfaction with the visit was measured (“very dissatisfied” to “very satisfied”). Qualitive analysis of an open‐ended comment question captured respondents’ perceptions about the visit.

**Results:**

Satisfaction surveys were completed by 338 out of 748 individuals (45%) over six months: 162 patients, 169 care partners, 7 did not specify. There were no statistically significant differences in ratings between patients and care partners. The majority of respondents were satisfied or very satisfied with their clinic visit (88%). The majority of respondents agreed or strongly agreed that they received an appointment in good time (95%), their questions were adequately addressed (93%), they were satisfied with the amount of time the team spent with them (95%), they had a better understanding of the condition (88%), they would recommend the memory clinic to others (94%), and the clinic was value added to their regular primary care (94%). Analysis of the open‐ended question identified that respondents valued having access to the clinic, the team approach, team members’ expertise and caring demeanor, and the comfortable assessment atmosphere.

**Conclusions:**

This study demonstrates high levels of patient and care partner satisfaction with MINT memory clinic care across 14 clinics in 4 provinces. This model meets the needs of individuals and families living with dementia and other memory disorders and offers a significant opportunity to build capacity within primary care for timely access to comprehensive, multispecialty memory assessment and care across Canada.

